# Biotechnological Strategies to Enhance Maize Resilience Under Climate Change

**DOI:** 10.3390/biology15020161

**Published:** 2026-01-16

**Authors:** Kyung-Hee Kim, Donghwa Park, Byung-Moo Lee

**Affiliations:** Department of Life Science, Dongguk University-Seoul, Seoul 04620, Republic of Korea; redanan@dongguk.edu (K.-H.K.); holly0328@naver.com (D.P.)

**Keywords:** abiotic stress, AI-powered phenotyping, biotechnology policy, climate change, climate resilience, CRISPR/Cas9, genomic selection, multi-omics, *Zea mays* L.

## Abstract

Maize (*Zea mays* L.), a vital crop for global food and economic security, faces intensifying biotic and abiotic stresses driven by climate change, including drought, heat, and erratic rainfall. This review synthesizes emerging biotechnology-driven strategies designed to enhance maize resilience under these shifting environmental conditions. We present an integrated framework that encompasses CRISPR/Cas9 and next-generation genome editing, Genomic Selection (GS), Environmental Genomic Selection (EGS), and multi-omics platforms—spanning transcriptomics, proteomics, metabolomics, and epigenomics. These approaches have significantly deepened our understanding of complex stress-adaptive traits and genotype-by-environment interactions, revealing precise targets for breeding climate-resilient cultivars. Furthermore, we highlight enabling technologies such as high-throughput phenotyping, artificial intelligence (AI), and nanoparticle-based gene delivery—including novel in planta and transformation-free protocols—that are accelerating translational breeding. Despite these technical breakthroughs, barriers such as genotype-dependent transformation efficiency, regulatory landscapes, and implementation costs in resource-limited settings remain. Bridging the gap between laboratory innovation and field deployment will require coordinated policy support and global collaboration. By integrating molecular breakthroughs with practical deployment strategies, this review offers a comprehensive roadmap for developing sustainable, climate-resilient maize varieties to meet future agricultural demands.

## Simple Summary

Maize is a cornerstone of global food security but faces escalating threats from climate change, including drought, heat waves, and unpredictable rainfall. This review highlights how cutting-edge biotechnologies are transforming maize breeding to meet these challenges. We explore the integration of CRISPR gene editing, genomic selection, and multi-omics approaches to develop climate-resilient varieties. Furthermore, we discuss enabling innovations such as AI-driven phenotyping and nanoparticle-mediated gene delivery that are accelerating the breeding process. By synthesizing these advances, this summary provides actionable insights for researchers and breeders striving to secure sustainable maize production in a changing world.

## 1. Introduction

**Figure 1:**
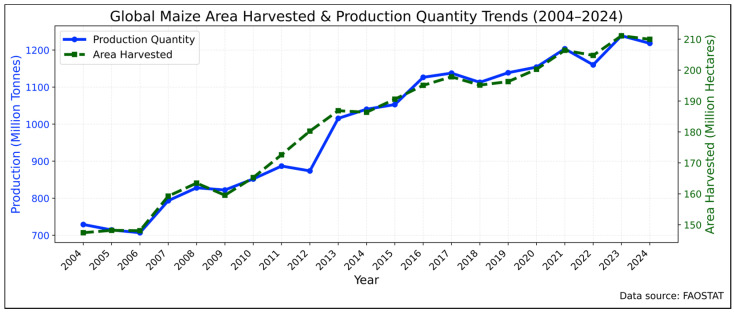
Global maize area harvested and production quantity trends from 2004 to 2024. The blue line indicates production (million tonnes), and the green dashed line indicates area harvested (million hectares). Data sourced from FAO [3].

Global climate change poses a significant threat to the sustainability of agriculture, with maize (*Zea mays* L.) being particularly susceptible to fluctuations in temperature and precipitation patterns [1,2]. Despite these challenges, global maize production has exhibited a robust upward trajectory. According to recent statistics, worldwide grain output increased from 729.0 million tonnes (Mt) in 2004 to 1,218.2 Mt in 2024, while the harvested area expanded from 147.4 million hectares (Mha) to 210.0 Mha over the same period (Figure 1) [3]. Notably, both production and harvested area reached their peaks in 2023—at 1,238.6 Mt and 211.1 Mha, respectively—followed by a slight decline in 2024. This substantial overall growth, where production increases have largely outpaced area expansion, underscores the critical role of yield improvements driven by modern breeding and agronomic advances.

However, these global gains mask significant regional disparities across climatic zones. Temperate regions, such as North America, East Asia, and parts of Europe, typically achieve the highest productivity (often >10 t/ha) through intensive hybrid-based systems. In stark contrast, subtropical and tropical regions in sub-Saharan Africa, Latin America, and South Asia—despite covering the majority of the global maize area—remain heavily reliant on rainfed systems where yields frequently stagnate below the global average, often remaining under 3–5 t ha^−1^, due to climate variability and limited inputs [4,5,6,7]. Recent assessments highlight that yield instability is most severe in these tropical environments, where recurrent droughts, heat waves, and erratic rainfall frequently coincide with low-input farming, exacerbating climate risks for smallholder farmers [8]. Furthermore, maize is cultivated in diverse varietal types, primarily classified into hybrids, open-pollinated varieties (OPVs), and composites, each possessing distinct breeding histories and climate sensitivities. Hybrids generally exhibit superior yield potential and stress resilience due to heterosis but typically require higher input levels and reliable annual seed replacement, making them dominant in temperate zones and commercial sectors of the tropics [6]. Conversely, OPVs and composite varieties—populations with broad genetic bases maintained by open pollination—remain crucial for marginal environments where farmers prioritize seed saving, local adaptation, and production stability over maximum yield [7]. Climate change is expected to differentially impact these groups: while hybrids may face significant physiological penalties if heat and drought stress occur during critical reproductive stages, OPVs and composites in tropical zones are increasingly threatened by combined abiotic and biotic stresses that compromise both yield and grain quality. This dichotomy underscores the urgent need for tailored climate-resilient improvement strategies that address the specific genetic and agronomic requirements of each varietal type [4].

Given that maize is a cornerstone of global food and economic security, ensuring the stability of its production is paramount. However, recent climate projections paint a sobering outlook: by the end of this century, global warming could reduce maize yields by 12–28%, even with extensive adaptation strategies in place [9]. An increase in temperature of just 1 °C is predicted to result in a global average yield reduction of 4.03–7.4%, with regional decreases of up to 25% anticipated by 2050 in major production regions such as the central United States. Furthermore, historical and future projections for North America emphasize the importance of breeding and management innovations. Nevertheless, under high-emission scenarios, the projected climate-driven yield losses may outpace the gains from adaptation [2]. Climate change exerts multifaceted impacts on maize throughout all developmental stages, resulting in significant agronomic and physiological disruptions [1,10]. Elevated temperatures accelerate phenological progression and impair key reproductive events, most notably by reducing pollen viability and hindering silk emergence, which compromises fertilization and kernel development [11]. Drought stress further aggravates these effects by diminishing photosynthetic capacity and carbon assimilation while inducing premature leaf senescence, ultimately limiting yield potential [11,12]. Additionally, the increasing frequency of extreme weather events, such as flash droughts and flooding, impairs crop establishment and exacerbates yield instability, particularly in vulnerable production systems [10]. These cumulative stressors underscore the urgent need for climate-resilient agronomic practices and breeding innovations to sustain maize productivity under escalating environmental volatility. Traditional breeding approaches, while foundational, are increasingly insufficient to keep pace with the rapid and complex requirements of climate resilience in maize [13,14]. This limitation has catalyzed the integration of advanced biotechnological tools—including molecular genomics, genome editing, and high-throughput phenotyping—into modern crop improvement programs [13,14,15]. Recent breakthroughs in technologies such as quantitative trait loci (QTL) mapping, genome-wide association studies (GWAS), and CRISPR/Cas9-based genome editing have enabled unprecedented precision in identifying, characterizing, and deploying genes that confer climate resilience [14,15]. The CRISPR/Cas9 system, in particular, has shown exceptional utility for targeted gene modification, facilitating the rapid improvement of traits such as drought tolerance, heat resistance, and complex abiotic stress responses. Integrating these molecular innovations with conventional breeding accelerates genetic gain and expands the adaptive capacity of maize in the face of intensifying climate variability [13,14]. Against this background, this review synthesizes recent advances in climate-resilient maize improvement, with particular emphasis on the deployment of genome editing and other modern biotechnological tools within maize breeding pipelines.

### Methodology and Data Collection

This review synthesizes recent advancements in biotechnological strategies for maize improvement, focusing primarily on literature published between 2020 and 2025 to capture the most rapidly evolving technologies. To ensure a comprehensive and systematic analysis, data were collected from major scholarly databases, including Web of Science, PubMed, Scopus, and Google Scholar. The literature search employed specific Boolean keyword combinations targeting three core domains: (1) climate change impacts (e.g., “maize climate resilience,” “drought and heat stress mechanisms”); (2) advanced breeding technologies (e.g., “CRISPR-Cas9 maize transformation,” “environmental genomic selection,” “high-throughput phenomics”); (3) emerging solutions (e.g., “nanoparticle-mediated delivery,” “multi-omics integration”). The selection process prioritized peer-reviewed original research articles and high-impact reviews that demonstrated breakthrough methodologies or provided empirical evidence of stress tolerance improvements. While the primary focus was on recent developments, foundational studies and seminal papers pre-dating 2020 were also included to provide essential historical context and theoretical framing. In total, approximately 129 publications were critically evaluated and selected based on their relevance to transforming maize breeding pipelines for climate adaptation. Furthermore, this review integrates insights from recent FAO statistical data [3] to ground the technical discussion in the reality of global production trends.

## 2. Climate Change Impacts on Maize Growth and Development

Climate change-induced abiotic stresses, particularly heat, drought, and erratic rainfall, adversely affect maize growth across all developmental stages. These stresses disrupt both vegetative and reproductive processes through tightly coupled physiological and molecular mechanisms, ultimately threatening yield stability [16,17].

Drought stress primarily impacts plant water status by reducing leaf water potential and turgor pressure. Physiologically, this water deficit triggers the rapid accumulation of abscisic acid (ABA) in roots, which signals stomatal closure in leaves to minimize transpiration, albeit at the expense of reduced CO_2_ assimilation and photosynthetic rate [16]. At the cellular level, severe dehydration leads to the overproduction of reactive oxygen species (ROS), such as superoxide radicals and hydrogen peroxide. These ROS cause oxidative damage to lipids, proteins, and DNA, thereby impairing cellular homeostasis and inhibiting kernel development [18]. Morphologically, maize plants respond by altering root architecture—often shifting from a dense, fibrous system to deeper but sparser rooting to access subsoil moisture—which can paradoxically reduce nutrient uptake efficiency in topsoils [16].

Heat stress imposes distinct but often overlapping challenges. It is particularly damaging during the reproductive phase; canopy temperatures exceeding 33–36 °C significantly compromise pollen viability and germinability due to the dehydration of silk tissues and the loss of membrane thermostability [17]. High temperatures disrupt the structural integrity of thylakoid membranes in chloroplasts, reducing the efficiency of Photosystem II and further diminishing carbon sources available for grain filling [18]. This stress often extends the anthesis–silking interval (ASI), causing asynchronous pollination and leading to substantial kernel abortion [17]. At the molecular level, heat stress induces the expression of heat shock proteins (HSPs) as molecular chaperones to protect proteins from denaturation and reprograms hormone signaling pathways, including auxin and ethylene, to modulate growth responses [18].

**Figure 2:**
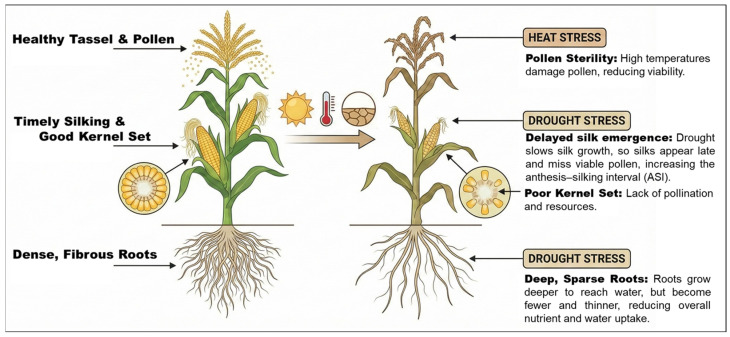
Physiological and morphological impacts of climate change-induced abiotic stresses on maize development. The diagram contrasts the developmental phenotypes of a healthy maize plant (**left**) with one subjected to heat and drought stress (**right**). (**Top**) Heat stress during anthesis directly damages male reproductive tissues, causing pollen sterility and reduced viability. (**Middle**) Drought stress retards silk growth, resulting in delayed silk emergence and an extended Anthesis–Silking Interval (ASI), which leads to poor kernel set due to asynchronous pollination. (**Bottom**) Under water-deficit conditions, the root system architecture is modified; roots grow deeper to access moisture but become fewer and thinner (sparse), compromising overall nutrient and water uptake efficiency.

In field conditions, these abiotic stresses frequently coincide with biotic pressures, forcing maize to balance survival-oriented stress responses with immune defense, often resulting in a trade-off that penalizes yield [16,17]. Figure 2 illustrates the major physiological and morphological impacts of heat and drought stress on maize, highlighting disruptions in tassel fertility, silk emergence, root development, and kernel set compared with optimal growth conditions.

### 2.1. Impacts on Morphological and Physiological Properties

Climate change fundamentally reshapes the morphological architecture and physiological function of maize, with temperature extremes and water deficits exerting particularly severe effects [1,9,10,11,12,19,20]. Recent empirical studies indicate that temperatures in the range of 33–36 °C during key reproductive processes, such as flowering and pollination, severely disrupt reproductive physiology; specifically, each 1 °C rise above 30 °C can reduce yields by 1–1.7% in warm, maize-dependent regions [2,9,10,19,21,22]. The reproductive phase is especially sensitive: temperatures above 35 °C during flowering markedly reduce pollen production and viability by up to 50%, while those exceeding 38 °C accelerate silk desiccation, ultimately lowering kernel set [19,21,22,23,24,25]. A hallmark physiological response to combined heat and drought is the extension of the anthesis–silking interval (ASI). Stress-induced delays in silk emergence relative to pollen shed (often by 1–2 days) disrupt pollen–silk synchrony, promoting kernel abortion and sharply reducing grain set [11,14,19,20,26,27]. Furthermore, elevated night-time temperatures increase respiratory carbon losses and deplete assimilate reserves, thereby impairing grain-filling efficiency and quality [12,19,25]. Conversely, low-temperature stress during early development impairs seed germination and seedling establishment, resulting in stunted growth and delayed flowering, which are major limitations in temperate climates [28,29,30,31].

Drought remains the dominant abiotic constraint, with annual yield losses often estimated between 20% and 40% [1,11,14,20]. While water deficit during vegetative growth restricts leaf expansion and stem elongation [11,12,26], the period spanning approximately two weeks before to two weeks after silking is most critical, potentially causing yield losses exceeding 50% under severe conditions [11,14,20,26,27]. Despite these challenges, breeding efforts have achieved genetic gains in water use efficiency (WUE)—approximately 0.305 kg ha^−1^ mm^−1^ per year—allowing modern genotypes to produce more grain per unit of water [2,32]. Root architecture responds dynamically to soil moisture: maize often invests in deeper rooting to access subsoil water under drought, whereas flooding induces root zone hypoxia and tissue necrosis, compromising nutrient uptake [10,26,27,30]. In parallel, warming modifies pest dynamics; invasive pests such as the fall armyworm (*Spodoptera frugiperda*) are expanding their overwintering ranges into previously cool regions, increasing the frequency of morphological damage to the crop [33,34].

### 2.2. Impacts on Biochemical Events

The visible morphological disturbances are underpinned by profound biochemical imbalances at the cellular and tissue levels, involving oxidative stress, osmotic regulation, and hormone-mediated trade-offs [1,11,12,13,14,28,29,30]. Heat, drought, and their combinations accelerate the generation of reactive oxygen species (ROS), such as hydrogen peroxide and superoxide radicals. Excessive ROS promote lipid peroxidation and membrane destabilization, evidenced by increased malondialdehyde (MDA) concentrations [11,12]. Under such conditions, stressed plants typically show increased electrolyte leakage and impaired photosystem efficiency. In response, tolerant genotypes more effectively upregulate antioxidant enzymes—such as superoxide dismutase (SOD), catalase (CAT), and peroxidases (POD)—to scavenge ROS and maintain redox homeostasis [11,12,13,28].

Osmotic adjustment is another central biochemical strategy. Stress triggers the accumulation of compatible solutes, including proline, glycine betaine, and soluble sugars, to maintain cell turgor and stabilize proteins [13,14,35]. For instance, the plasma-membrane protein ZmPMP3g enhances drought resilience by regulating the balance between abscisic acid (ABA) and gibberellic acid (GA), increasing antioxidant enzyme activities, and facilitating osmolyte accumulation [35]. However, hormonal regulation introduces critical trade-offs. Water deficit strongly induces ABA accumulation to promote stomatal closure, yet ABA-driven pathways can antagonize salicylic acid (SA) and jasmonic acid (JA) signaling networks essential for immune defense [13,14,36,37,38]. Consequently, plants that favor abiotic stress tolerance may rely on ABA-mediated survival at the expense of immune activation, increasing vulnerability to pathogens [19,36,37,38]. Additionally, warmer and more humid conditions favor the proliferation of mycotoxigenic fungi (*Fusarium* and *Aspergillus* spp.), leading to the biochemical accumulation of aflatoxins and fumonisins in grain, which poses severe safety challenges [39,40,41].

### 2.3. Impacts on Biomolecular Mechanisms

Morphological and biochemical responses are orchestrated by complex biomolecular mechanisms integrating stress perception and transcriptional regulation [1,13,14,19,26]. Transcriptomic analyses reveal extensive reprogramming of gene expression, including the strong induction of Heat Shock Protein (HSP) families—such as HSP20, HSP70, and HSP101—and diverse stress-responsive transcription factors (TFs) from the WRKY, MYB, bZIP, and NAC families [19,23,25,42]. These HSPs act as molecular chaperones to stabilize denatured proteins, while the TFs coordinate downstream networks controlling metabolic reprogramming [19,23,42]. Comparative studies consistently show that heat-tolerant genotypes exhibit earlier and stronger induction of these protective genes compared to sensitive lines [10,13,17].

The genetic architecture of climate resilience is highly polygenic. QTL and association studies have identified key genomic regions controlling traits such as root architecture, ASI, and water-use efficiency, highlighting loci such as *Dwarf8* and *Vgt2* as pivotal for climate adaptation [14,26,27,31]. Furthermore, recent research highlights the contribution of long non-coding RNAs (lncRNAs) and alternative splicing to stress adaptation. For example, under cold and drought stress, these molecules regulate mRNA stability and protective responses against photoinhibition and oxidative damage in roots and emerging leaves [28,29,30]. Understanding how these transcriptional regulators and signaling components integrate multiple environmental cues is essential for designing maize genotypes that maintain both abiotic stress resilience and biotic resistance [13,19,36,37,38].

### 2.4. Critical Physiological and Molecular Changes Under Abiotic and Biotic Stress

Across abiotic stresses such as drought, heat, chilling, flooding, and salinity, maize exhibits a characteristic suite of physiological and molecular alterations at the whole-plant, tissue, and cellular levels. These include reduced photosynthetic capacity due to chlorophyll degradation, perturbed water relations, and increased oxidative damage from the excessive accumulation of reactive oxygen species (ROS) [43,44]. The imbalance between ROS production and scavenging capacity is a critical determinant of damage severity. To counteract these effects, maize initiates adaptive responses such as the accumulation of osmolytes (e.g., proline) and the activation of antioxidant defense enzymes like superoxide dismutase (SOD) and peroxidase (POD) [43]. Reproductive processes are particularly sensitive to these shifts. Stress-induced changes lead to extended anthesis–silking intervals (ASI), reduced pollen viability, impaired silk emergence, and disrupted endosperm development. Collectively, these changes result in poor kernel set and yield instability [17]. On a molecular level, these responses are governed by complex shifts in hormone balances, predominantly the accumulation of abscisic acid (ABA), which triggers stomatal closure but can also inhibit growth-related signaling [43].

**Figure 3:**
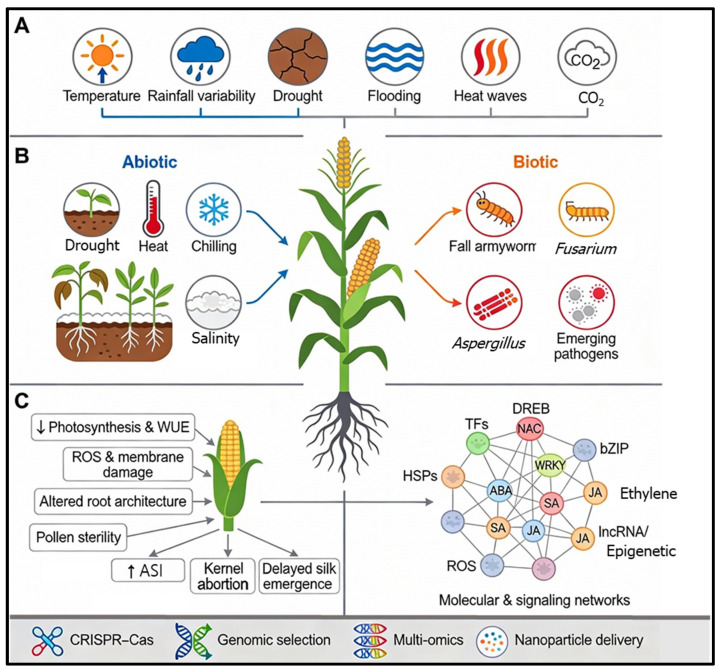
Hierarchical impacts of climate change on maize: From environmental drivers to molecular reprogramming. (**A**) Major climate change drivers, including elevated temperature, rainfall variability, and rising CO_2_, intensify agricultural risks. (**B**) These drivers trigger a cascade of abiotic stresses (drought, heat, chilling, salinity) and biotic pressures (insect pests like fall armyworm, fungal pathogens, and emerging diseases). (**C**) At the whole-plant level (**left**), these stresses compromise physiological and reproductive traits, leading to reduced photosynthesis, altered root architecture, pollen sterility, and an extended anthesis–silking interval (ASI). At the molecular level (**right**), maize responds through complex signaling networks involving transcription factors (e.g., DREB, NAC, WRKY), hormone crosstalk (ABA, SA, JA, ethylene), heat shock proteins (HSPs), and epigenetic modifications. The figure also highlights key leverage points where advanced biotechnological strategies, including CRISPR-based genome editing, genomic selection, multi-omics integration, and nanoparticle-mediated delivery, can be applied to enhance climate resilience in maize (further discussed in Section 3, Section 4 and Section 5). In Panel (**B**), blue arrows indicate the impact of abiotic stresses, while orange arrows represent the pressure from biotic stressors. In Panel (**C**), arrows illustrate the physiological consequences leading to the activation of molecular signaling networks.

Concurrently, climate-driven changes in temperature and moisture alter the geographic range and severity of biotic threats. Insect pests and mycotoxigenic fungi, such as fall armyworm (*Spodoptera frugiperda*), *Fusarium* spp., and *Aspergillus* spp., pose increasing risks to grain yield and quality [17]. A critical challenge arises when abiotic and biotic stresses coincide; maize must manage the antagonistic crosstalk between ABA-mediated drought adaptation and salicylic acid (SA)/jasmonic acid (JA)-dependent immune competence [43]. This trade-off often compromises the plant’s ability to mount robust immune responses while conserving water. An integrated overview of these hierarchical impacts—from environmental drivers to molecular reprogramming—is illustrated in Figure 3. Furthermore, a comprehensive summary of these major stress factors, along with their specific physiological impacts and molecular mechanisms identified in recent research, is presented in Table 1. Understanding these critical changes provides the biological foundation for deploying advanced biotechnological interventions. Technologies such as CRISPR-based editing, genomic selection, and nanoparticle-mediated delivery systems are being actively developed to target these stress-responsive pathways [45,46]. By precisely manipulating these molecular regulators, researchers aim to develop maize genotypes that maintain high yield and grain quality under increasingly variable and hostile climates.

## 3. Biotechnological Approaches for Climate Resilience

**Figure 4:**
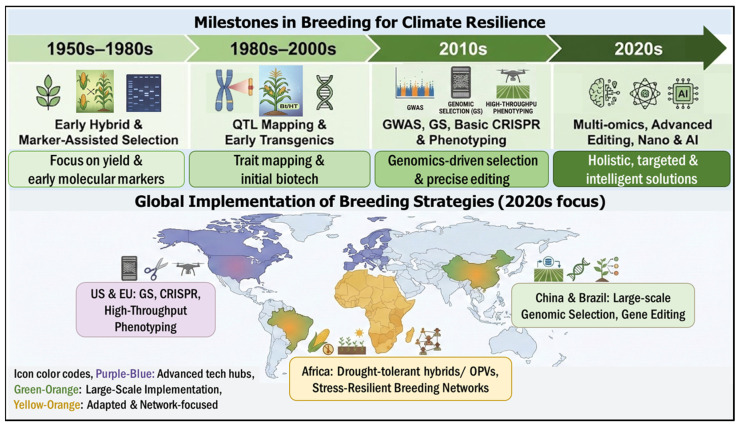
Timeline of maize breeding innovations and global implementation strategies for climate resilience. The upper panel illustrates the evolution of breeding technologies from the mid-20th century to the present, highlighting the transition from early hybrid development and QTL (Quantitative Trait Loci) mapping to contemporary genomic selection (GS), CRISPR-Cas genome editing (including base and prime editing), and the integration of multi-omics with AI/ML (Artificial Intelligence/Machine Learning). The arrow at the bottom indicates the trend toward increasing precision and technological integration. The lower panel depicts the global landscape of these strategies in the 2020s, categorized by regional focus: advanced technological hubs in the US and EU (purple-blue), large-scale implementation of GS and gene editing in China and Brazil (green-orange), and the deployment of drought-tolerant varieties and stress-resilient breeding networks in Africa (yellow-orange).

The trajectory of maize improvement has undergone a transformative shift over the past quarter-century, evolving from empirical phenotypic selection to precision, data-driven breeding systems. As visually summarized in Figure 4, this evolution is characterized by pivotal milestones ranging from the early hybrids and QTL mapping of the 20th century to the routine implementation of Genomic Selection (GS) and high-throughput phenotyping in the 2010s. Most recently (2020–2025), the integration of CRISPR–Cas9 genome editing and AI-driven multi-omics has further accelerated this progress. To provide the necessary historical and geographical context for the advanced strategies discussed in this review, Figure 4 chronicles these major biotechnological milestones and illustrates the global landscape of their implementation. This roadmap highlights how the progressive convergence of genomics, phenomics, and biotechnology has laid the foundation for the current era of climate-smart maize breeding. Enhancing abiotic stress resilience in maize requires the systematic integration of genomic discovery and biotechnological manipulation. As outlined in the historical milestones in Figure 4, this strategy begins with deciphering the genetic architecture of complex traits and culminates in the precise engineering of key genomic targets. In this section, we discuss the identification of stress-responsive genomic regions through quantitative trait loci (QTL) mapping and genome-wide association studies (GWAS) (Section 3.2 and Section 3.3). We then examine how these targets are functionally validated and improved using advanced biotechnological interventions, specifically CRISPR/Cas9 gene editing, transgenic approaches, and nanotechnology-mediated delivery (Section 3.4, Section 3.5 and Section 3.6).

### 3.1. Evolution of Breeding Frameworks: From Conventional to Environmental Genomic Selection

#### 3.1.1. Conventional Maize Breeding Cycles: OP, Hybrid, and Composite

**Figure 5:**
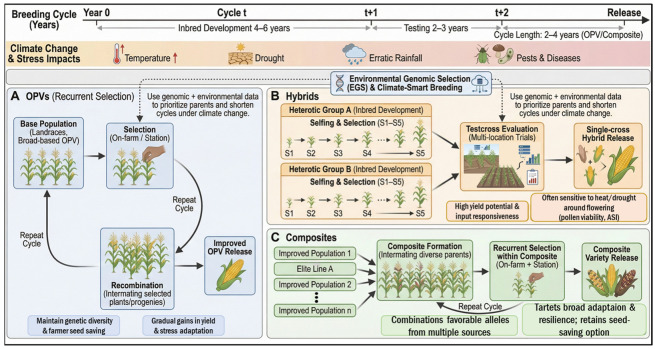
Conventional maize breeding cycles and their integration with Environmental Genomic Selection (EGS) for climate adaptation. The diagram illustrates the chronological breeding timelines and procedures for different maize types under climate stress impacts. Red arrows in the top panel indicate the increasing intensity of climate stress factors. (**A**) Open-Pollinated Varieties (OPVs): Shows recurrent selection to improve yield while maintaining genetic diversity and enabling farmer seed saving. (**B**) Hybrids: Depicts the development of inbred lines (S1–S5) from distinct heterotic groups, followed by testcross evaluation for high yield and uniformity, noting potential sensitivity to stress. (**C**) Composites: Illustrates formation by intermating diverse improved populations or elite lines, followed by selection for broad adaptation and resilience, retaining the seed-saving option. The central box highlights how Environmental Genomic Selection (EGS), utilizing genomic and environmental data, is integrated (dashed arrows) to prioritize parents and shorten breeding cycles in response to climate change, while solid arrows represent the sequential progression of breeding stages.

Prior to the advent of high-throughput molecular tools, maize improvement relied predominantly on conventional breeding cycles centered on phenotypic selection. To visualize the structural differences and modern adaptation of these methodologies, Figure 5 provides a schematic comparison of the three primary breeding cycles. As illustrated in Panel A, Open-Pollinated Varieties (OPVs) utilize recurrent selection to improve population performance while preserving genetic variability, a critical feature for farmer seed systems. In contrast, Panel B details the rigorous process of Hybrid breeding, involving the development of inbred lines (S1–S5) within distinct heterotic groups to maximize yield potential through heterosis. Panel C depicts Composite formation, where diverse elite lines are intermated to create populations with broad environmental adaptability. Crucially, the diagram highlights the contemporary integration of Environmental Genomic Selection (EGS) (top dash-lined box), demonstrating how genomic and environmental data are now overlaid onto these traditional cycles to accelerate parental selection and shorten the breeding timeline in response to climate change.

Historically, maize cultivation depended on Open-Pollinated Varieties (OPVs), which were maintained by farmers through mass selection of visually superior ears. While this practice preserved high genetic diversity and facilitated adaptation to local, low-input environments, OPVs generally exhibited lower yield potential due to the limited exploitation of heterosis [7,47]. The subsequent transition to Hybrid Breeding fundamentally transformed maize production. By developing homozygous inbred lines through repeated selfing and crossing of them to produce uniform *F*_1_ hybrids, plant breeders were able to harness hybrid vigor (heterosis), resulting in substantial gains in grain yield, uniformity, and stability that underpin modern intensive agriculture [6,48]. To reconcile the genetic uniformity of hybrids with the broad adaptability of OPVs, Composite and Synthetic Varieties were developed through the intermating of diverse germplasm sources. These dynamic populations function as reservoirs of allelic diversity, maintaining the evolutionary potential required for adaptation to increasing climatic variability [6,47]. Despite their historical and practical significance, these conventional cycles face substantial limitations in the context of rapid climate change. They are inherently time-consuming—often requiring 5–8 years per cycle from initial crosses to variety release—and their efficiency remains constrained by pronounced genotype-by-environment (G × E) interactions, which are difficult to resolve without extensive, multi-year, multi-location field testing [6,49].

#### 3.1.2. Environmental Genomic Selection (EGS) for Climate-Smart Breeding

To overcome the inherent limitations of phenotypic selection, Genomic Selection (GS) has transformed maize breeding by utilizing genome-wide marker data to predict genomic estimated breeding values (GEBVs). This approach decouples selection decisions from direct phenotypic evaluation, enabling the early identification of superior lines and significantly shortening breeding cycles [50,51]. However, standard GS models typically treat environments as fixed or random factors without explicitly accounting for specific environmental drivers. This limitation often reduces prediction accuracy when genotypes are evaluated under contrasting or novel climate scenarios, as the models fail to capture the underlying causes of genotype-by-environment (G × E) interactions [52]. Environmental Genomic Selection (EGS) extends the standard GS framework by incorporating explicit environmental covariates—such as temperature dynamics, precipitation regimes, vapor pressure deficit, and soil physicochemical properties—directly into genomic prediction models [53,54]. By explicitly modeling *G* × E interactions using reaction norm models or by integrating process-based crop growth models, EGS enables breeders to predict genotype performance under plausible future environmental scenarios rather than relying solely on historical environmental averages [52]. Recent studies demonstrate that EGS and envirotyping-based approaches can substantially improve prediction accuracy across heterogeneous agro-ecological zones, serving as a vital bridge between conventional population improvement and site-specific, climate-smart adaptation strategies [51,54].

### 3.2. Quantitative Trait Loci (QTL) Mapping and Marker-Assisted Selection

Quantitative trait locus (QTL) mapping continues to be a pivotal tool for dissecting the genetic basis of complex climate-resilience traits in maize [26]. Technological advancements have greatly improved the resolution and robustness of QTL detection, enabling the identification of genomic regions governing drought and heat tolerance, as well as yield stability. A recent meta-QTL analysis synthesized 56 QTL datasets and identified 83 meta-QTLs (MQTLs) associated with drought and yield-related traits distributed across the maize genome [55]. Among them, five major MQTL clusters located on chromosomes 1, 2, 5, 6, and 8 are summarized in Table 2. These consensus regions exhibit narrow confidence intervals and frequently co-localize with multi-trait QTLs, highlighting their pleiotropic effects and stable expression across diverse environments [55,56].

One of the most successful applications of these discoveries involves the Anthesis–Silking Interval (ASI), a critical determinant of kernel set under water deficit. Stable QTLs for ASI have been consistently mapped to chromosomes 1, 2, and 6 [26,56]. Maize breeding programs have incorporated molecular markers linked to these QTLs, resulting in commercial varieties with significantly improved synchronization between pollen shed and silk emergence. Similarly, root architecture traits have been targeted to enhance resource capture. Meta-analyses have revealed genomic “hotspots,” such as umc11 (chr1) and csu133 (chr2), with confidence intervals often below 1 Mb, making them promising candidates for allele mining [55]. Varieties incorporating favorable alleles for root depth and angle have demonstrated improved water and nitrogen use efficiency, a trait particularly valuable for ensuring productivity in rainfed systems. To validate these loci, researchers are increasingly utilizing Near-isogenic lines (NILs) and high-throughput platforms before deploying them into breeding populations. Marker-assisted selection (MAS) has substantially accelerated genetic progress by enabling breeders to efficiently introduce these favorable alleles into elite hybrids. This value is best demonstrated by the development of drought-tolerant maize varieties for sub-Saharan Africa. Through international collaboration, QTLs identified in temperate germplasm were introgressed into tropical varieties, achieving yield advantages of 20–30% under drought stress while maintaining competitive yields in favorable conditions. Modern MAS platforms now integrate tightly linked DNA markers and genome-based selection algorithms to track these complex traits. Between 2021 and 2025, the combined application of MAS and genomic selection (GS) has facilitated the rapid deployment of climate-resilient cultivars [26,57]. Moreover, multi-locus and omics-driven MAS strategies are increasingly being implemented to pyramid QTLs conferring multiple stress responses, thereby improving both the efficiency and precision of climate-smart maize breeding programs [57,58].

### 3.3. Genome-Wide Association Studies (GWASs)

Genome-wide association studies (GWASs) have emerged as a powerful complement to traditional QTL mapping, providing higher resolution for dissecting the genetic architecture of complex climate resilience traits in maize [26]. The utilization of large, diverse maize populations and high-density SNP genotyping platforms has allowed for the identification of numerous loci associated with drought tolerance, heat resistance, and stable yield under stress [59,60]. For instance, a comprehensive GWAS involving 379 inbred maize lines identified 15 candidate genetic variants significantly associated with seedling drought resistance [59]. These SNPs mapped to genes crucial for metabolism, programmed cell death, transcriptional regulation, and autophagy. Recent functional validation has further characterized genes such as *ZmGRAS15*—a core regulator of root length and drought adaptation—and others like *ZmDREB2.5* and *ZmAREB*, accelerating the discovery of functionally relevant alleles for breeding [26,59]. While GWASs effectively highlight potential genomic targets, translating these insights into tangible stress tolerance often requires precise validation and manipulation. This bridge between genomic discovery and functional genomics paves the way for targeted engineering technologies such as CRISPR/Cas9, as discussed in the following section.

### 3.4. CRISPR/Cas9 Gene Editing

The emergence of CRISPR/Cas9 technology has fundamentally transformed the field of crop improvement, providing plant breeders with the unprecedented ability to execute precise, targeted genomic modifications, often without the permanent integration of foreign DNA [15,61]. This platform is now established as a cornerstone for developing climate-resilient maize, enabling the direct editing of genes governing abiotic and biotic stress tolerance, yield stability, and complex adaptive traits essential for agriculture under changing climatic conditions. Successful applications have demonstrated the efficacy of this approach in real-world scenarios. A prominent example is the targeted editing of the *ZmARGOS8* promoter to enhance drought tolerance. By replacing the native promoter or inserting a constitutive *GOS2* promoter sequence into the 5′ untranslated region, researchers significantly elevated gene expression. Multi-location field trials confirmed that these edited lines achieved yield improvements of up to five bushels per acre under drought conditions, with no yield penalty in well-watered environments [61,62]. Similarly, CRISPR/Cas9-mediated modification of *SlAGL6* (*SlAGamous-Like 6*) has been shown to enhance resilience to heat stress during critical growth stages [15]. Furthermore, recent advancements in base editing and multiplex CRISPR strategies have facilitated the simultaneous editing of multiple genes, accelerating the development of elite cultivars. Significant progress includes the generation of null mutants in *ZmGDIα*, which conferred robust resistance to maize rough dwarf disease (MRDD) without agronomic penalties, and the modification of *ZmCOIα* and *ZmCCT* for improved disease resistance and yield potential. Other notable applications involve *ZmSRL5* for strengthened cuticular wax and drought tolerance, *ZmCLCg* for salt stress resilience, and *ZmACC1/2* for herbicide resistance [15,63]. These cases collectively underscore the power of integrating precision gene editing with rigorous field-based validation to ensure sustainable agricultural productivity in increasingly challenging environments.

#### 3.4.1. Transgene-Free and DNA-Free Genome Editing in Maize

Beyond the precision of the edits themselves, the methodology of delivery plays a pivotal role in practical application. In agricultural biotechnology, “transgene-free” genome editing denotes the generation of progeny without stably integrated foreign DNA, a concept closely aligned with “DNA-free” editing, wherein CRISPR machinery is delivered as preassembled ribonucleoproteins (RNPs) or RNA [18]. This approach ensures that only targeted indels or precise allelic changes are retained, avoiding any exogenous genetic footprint [18,64]. Adopting such transgene-free strategies is critical for climate-resilient maize breeding. It significantly minimizes regulatory burdens and public concerns regarding GMOs while reducing the risk of long-term off-target effects associated with constitutive transgene expression [18,65]. Various platforms have been optimized for maize to achieve this, notably polyethylene glycol (PEG)-mediated RNP delivery. Research demonstrates that PEG–calcium transfection in maize leaf protoplasts can induce targeted mutations at frequencies of 0.85–5.85% within 15 days, proving the technical viability of DNA-free delivery even in recalcitrant species [64]. Looking forward, the integration of these platforms with nanoparticle-based and in planta delivery strategies promises to accelerate the development of elite lines. By enabling the modification of stress-tolerance loci without leaving selectable marker cassettes, these technologies facilitate rapid deployment in climate-vulnerable regions [17,65]. Ultimately, these methods complement conventional genomic selection, providing a precise mechanism to introduce favorable alleles into adapted local germplasm while maintaining essential genetic diversity [17].

#### 3.4.2. Protoplast-Based CRISPR–Cas Delivery Systems in Maize

Protoplast-based systems serve as a critical intermediate platform for validating CRISPR designs, bridging the gap between in silico target selection and stable transformation. This process typically begins with isolating viable protoplasts from leaf mesophyll or embryogenic cultures using optimized enzymatic digestion (cellulase and macerozyme). By employing robust polyethylene glycol (PEG)–Ca^2+^ protocols, researchers can efficiently deliver Cas9–sgRNA ribonucleoproteins (RNPs) into isolated protoplasts [64]. This system allows for the precise quantification of editing efficiency via amplicon sequencing or T7E1 assays within just 10–15 days, providing a rapid feedback loop to balance transfection efficiency with cell viability [18,64]. Importantly, these systems have evolved beyond simple indel detection into functional assays for evaluating loci associated with abiotic stress tolerance and nutrient-use efficiency before committing to time-consuming stable transformation processes [17]. Since many elite inbred lines remain recalcitrant to traditional *Agrobacterium* or biolistic transformation, protoplast assays provide a genotype-flexible chassis. This flexibility enables the benchmarking of emerging delivery chemistries, including nanoparticle-assisted delivery and polymer-based carriers, across diverse germplasm panels [65,66]. Although regenerating whole plants directly from protoplasts remains challenging due to genotype dependence and somaclonal variation, this system plays a vital strategic role. It significantly de-risks the deployment of DNA-free platforms by allowing for the rapid, low-cost optimization of RNP composition and dosage. Consequently, protoplast-based transfection has become an indispensable step in climate-resilient breeding pipelines, ensuring that only the most promising targets and constructs advance to field validation [17,18].

#### 3.4.3. Next-Generation Precision Editing: Base, Prime, and Multiplexing

**Figure 6:**
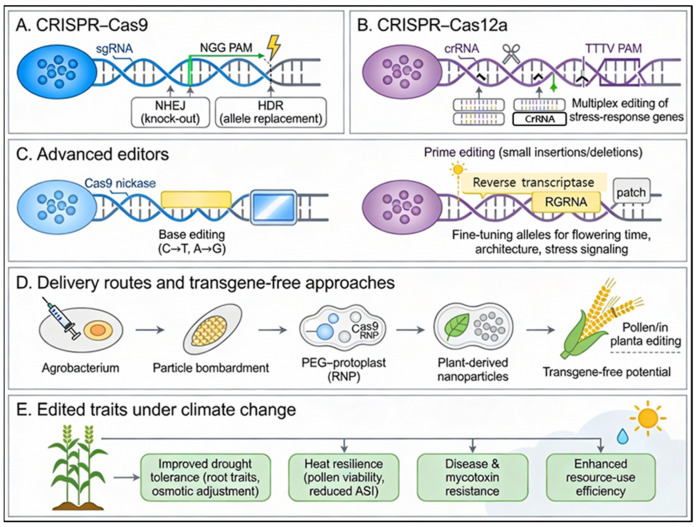
Mechanistic framework of CRISPR–Cas9 and Cas12-based strategies for developing climate-resilient maize. (**A**) The CRISPR–Cas9 system utilizes a single guide RNA (sgRNA) to direct the Cas9 nuclease to specific genomic loci flanked by an NGG protospacer adjacent motif (PAM). This induces double-strand breaks, which are repaired via error-prone non-homologous end joining (NHEJ) to create gene knock-outs or, less frequently, via homology-directed repair (HDR) for precise allele replacement. (**B**) CRISPR–Cas12a recognizes a distinct T-rich PAM (TTTV) and generates staggered cuts. This feature facilitates multiplex genome editing, enabling simultaneous modification of gene families and regulatory elements involved in complex stress-response networks. (**C**) Advanced editors, such as base editors (**left**) and prime editors (**right**), expand the genomic toolkit. Base editors use Cas9 nickase to induce targeted nucleotide substitutions (e.g., C → T, A → G), while prime editors employ a reverse transcriptase to mediate small insertions or deletions. These precise edits allow for the fine-tuning of alleles controlling traits like flowering time and plant architecture without generating double-strand breaks. (**D**) Diverse delivery systems are illustrated, including Agrobacterium-mediated transformation, particle bombardment, and transgene-free approaches such as PEG-mediated transfection of protoplasts with Cas9 ribonucleoproteins (RNPs) and plant-derived nanoparticles. (**E**) These integrated CRISPR strategies enable the precise improvement of agronomic traits, including drought and heat tolerance, disease resistance, and resource-use efficiency, to adapt maize to future climate scenarios. In the diagram, arrows indicate the step-by-step workflow of genome editing and repair processes, while different colors distinguish key molecular components such as Cas nucleases, guide RNAs, and target DNA strands.

Current advancements in CRISPR technology have evolved beyond simple knock-outs to high-precision modifications. Base editing technologies—utilizing optimized cytosine and adenine base editors (CBEs and ABEs)—now enable targeted single-nucleotide conversions without inducing double-strand breaks (DSBs), thereby significantly reducing off-target effects [63]. A prominent application of this technology is the precise editing of *ZmACC1* and *ZmACC2* genes to confer herbicide resistance. Researchers have achieved editing efficiencies of up to 100% at these loci, producing maize lines that support effective weed management in conservation tillage systems while maintaining normal yield potential. Furthermore, prime editing represents a next-generation leap, facilitating sophisticated modifications—including precise insertions, deletions, and base substitutions—without the need for donor templates. Although still emerging, this platform holds great promise for creating novel alleles previously unfeasible with traditional tools [67]. Concurrently, the rapid advancement of multiplexed gene editing allows for the simultaneous modification of multiple traits, addressing the polygenic nature of climate resilience. For instance, the combinatorial editing of targets such as *ZmGA20ox3*, *ZmSRL5*, *ZmACC1/2*, and *ZmSWEET1b* has successfully generated lines with “stacked” improvements. These include strengthened cuticular wax formation (*ZmSRL5*), enhanced salt resilience (*ZmCLCg*), and improved yield stability [63,68]. This capability enables breeders to aggregate beneficial alleles into elite germplasm within a single, efficient breeding cycle, accelerating the development of multi-stress tolerant cultivars (Figure 6).

#### 3.4.4. Limitations and Challenges of CRISPR-Based Editing for Climate-Resilient Maize

Despite the transformative potential of CRISPR technology for developing climate-resilient maize, several technical, biological, and socio-regulatory hurdles remain that must be carefully addressed in breeding pipelines [17]. First, off-target mutations are a primary concern, particularly in transformation systems where Cas nucleases are constitutively expressed. To ensure genomic integrity in elite germplasm, rigorous sgRNA design using dedicated in silico tools must be coupled with strict validation through whole-genome or targeted deep sequencing [17,69,70]. Second, biologically, the polygenic nature of climate-related traits—such as drought tolerance and pest resistance—means that single-gene edits often yield modest or context-dependent results. Consequently, integrating genome editing with genomic selection (GS), environmental genomic selection (EGS), and multi-omics is crucial to capture small-effect loci and resolve complex genotype-by-environment interactions [17,42]. Third, operationally, maize transformation remains highly genotype-dependent and resource-intensive. This creates a significant barrier to adoption for breeding programs with limited tissue-culture infrastructure [17,71,72]. Furthermore, heterogeneous regulatory frameworks and a lack of technological capacity, particularly in low- and middle-income regions, impede the global deployment of edited cultivars [73,74]. Overcoming these barriers requires innovations in transformation-free delivery platforms, such as DNA-free RNP, nanoparticle-, and pollen-mediated systems, which can bypass classical tissue culture [65,75]. These technical advances must be accompanied by sustained investment in biosafety assessment and transparent public engagement to secure societal acceptance and enable the equitable deployment of climate-resilient maize [17].

### 3.5. Transgenic Approaches and Gene Expression Modulation

Transgenic technology continues to play a vital role in developing climate-resilient maize varieties, particularly for traits that are governed by single or a few major genes and are difficult to improve through conventional breeding methods [76]. Recent advances in transgene design, promoter engineering, and gene stacking have significantly enhanced the effectiveness and precision of transgenic approaches for stress tolerance improvement [13,35]. A prime example of this success is the development of transgenic maize lines expressing the *ZmPMP3g* gene for improved drought tolerance. Overexpression of the *ZmPMP3g* gene has been shown to enhance drought tolerance through a series of interconnected mechanisms, including improved root growth, enhanced antioxidant capacity, and optimized abscisic acid–gibberellin (ABA-GA) hormone balance [35]. Under water-deficit conditions, these transgenic lines exhibited significantly higher leaf relative water content and a lower leaf wilting index, as well as increased total root length, compared to control plants. This demonstrates the practical utility of this approach.

Advanced transgenic strategies now allow much more precise control of gene expression in maize. First, promoter engineering plays a central role. Stress-inducible promoters activate tolerance-related genes only when the plant is exposed to drought, heat, or other stresses. This targeted activation optimizes resource allocation and prevents unnecessary fitness costs during normal growth conditions [13]. In addition, tissue-specific and synthetic promoters restrict gene expression to particular plant parts, such as roots or leaves. This ensures that the introduced genes act exactly where they are most effective, while minimizing unintended effects on overall plant development [76]. Second, gene stacking strategies have become increasingly important for building multi-stress tolerance. Modern approaches combine multiple genes—for drought tolerance, heat resistance, and pathogen resistance—into a single transgenic event. These so-called “pyramided” lines provide broad-spectrum protection against multiple stresses and consistently show stronger resilience in diverse field conditions [15]. Finally, the integration of these advanced transgenic tools with complementary methods such as genomic selection and CRISPR/Cas9-based editing offers enormous potential. Together, they form a powerful toolkit for developing climate-smart maize cultivars that can thrive under future climate challenges.

### 3.6. Nanotechnology-Mediated Gene Delivery Systems

Traditional maize transformation methods, such as *Agrobacterium*-mediated delivery and particle bombardment, have been instrumental for functional genomics and transgenic trait deployment but suffer from several intrinsic bottlenecks, including strong genotype dependence, low and variable transformation efficiencies, tissue damage, and labor-intensive, time-consuming tissue culture and regeneration steps. These limitations are particularly acute in many elite inbred lines and farmer-preferred cultivars that remain recalcitrant to current transformation protocols, thereby slowing the introgression of novel climate-resilience traits.

Recent advances in plant nanobiotechnology offer a disruptive alternative, providing species-independent, modular, and potentially high-throughput platforms for biomolecule delivery [77,78]. A wide range of nanomaterials—including mesoporous silica nanoparticles (MSNs), carbon nanotubes (CNTs), layered double hydroxide (LDH) particles, gold nanoparticles, and magnetic nanoparticles—have been engineered to transport plasmid DNA, RNA, small interfering RNAs, or CRISPR/Cas9 ribonucleoprotein (RNP) complexes across the rigid plant cell wall and plasma membrane with minimal tissue damage and without the need for external physical force such as biolistics or high-pressure infiltration [79,80,81]. For example, functionalized high-aspect-ratio CNTs can deliver plasmid DNA into intact leaves of both dicot and monocot species, achieving high transient expression without stable transgene integration and with broad host-species compatibility. Similarly, MSNs and related porous nanocarriers have been used to deliver genome-editing cargos, including proteins and RNPs, thereby enabling transgene-free editing pipelines that avoid foreign DNA integration. A particularly promising development for cereals is pollen-mediated transformation using magnetic nanoparticles, often termed “pollen magnetofection.” In this approach, exogenous DNA or CRISPR/Cas reagents are conjugated to superparamagnetic nanoparticles and introduced into freshly collected pollen grains under a magnetic field. Transfected pollen is then used directly for pollination, allowing delivery of genetic cargo into the germline and transmission to the progeny, while largely bypassing conventional callus induction and regeneration. Recent work in maize has demonstrated that optimization of particle size, surface chemistry, incubation temperature, and magnetic field parameters can substantially improve the efficiency, stability, and reproducibility of pollen magnetofection, making it an attractive route to genotype-independent, field-deployable transformation systems [77,78,81].

Another frontier is organelle-targeted gene delivery. Nanomaterials can be rationally functionalized with targeting peptides or ligands that enable selective accumulation in chloroplasts or mitochondria, opening new possibilities for precise editing of organellar genomes that are not accessible via conventional breeding [82,83,84]. Chloroplast-targeted CNTs and carbon dots, for instance, have been used to deliver plasmid DNA and agrochemicals into a large proportion of chloroplasts in mature leaves, achieving high transient expression with minimal cytotoxicity, and illustrating how organelle-specific delivery can be leveraged for photosynthesis engineering and stress-tolerance traits. When combined with CRISPR/Cas RNP cargoes, nanocarriers provide a flexible chassis to control cargo release, protect nucleoprotein complexes, and potentially enhance homology-directed repair, addressing several key challenges in plant genome editing [81,85,86]. By integrating these nanotechnology-based delivery systems with CRISPR and other next-generation editing platforms, researchers can significantly broaden the range of maize genotypes amenable to precise genetic modification, reduce dependence on somatic tissue culture, and accelerate breeding cycles for climate-resilient traits. In the longer term, nano-enabled, tissue-culture-free editing in pollen, embryos, or vegetative tissues could support decentralized, field-proximal transformation pipelines and facilitate more equitable access to advanced biotechnologies in regions where conventional transformation infrastructure is limited.

#### 3.6.1. Plant-Derived Nanoparticles as Delivery Platforms for CRISPR–Cas Systems

Plant-derived nanoparticles and biopolymers are emerging as sustainable and green (bio-based and environmentally benign) candidates for delivering CRISPR–Cas components into plant cells. Prominent examples include cellulose nanocrystals (CNCs), lignin-based nanocarriers, and zein protein particles. These materials are highly biocompatible and offer a safer alternative to synthetic nanomaterials for plant transformation. To ensure effective delivery, these nanocarriers undergo chemical functionalization. For instance, cationic modification with agents such as EPTMAC endows CNCs and zein with a positive surface charge. This allows them to bind negatively charged DNA, RNA, or ribonucleoprotein (RNP) complexes through electrostatic attraction, effectively condensing and protecting the molecular cargo [87]. Their nanoscale dimensions and tunable surface charge are essential for traversing the complex barriers of the plant cell wall and plasma membrane. Furthermore, the nanoparticle corona acts as a vital shield. It protects nucleic acids and proteins from degradation by extracellular nucleases and other hostile factors within the apoplast [77,88].

Recent proof-of-concept studies have validated this potential. Cationic CNC and zein nanoparticles, loaded with GFP-encoding plasmid DNA, successfully penetrated tobacco leaves through syringe injection or vacuum infiltration. These experiments demonstrated that plant-derived nanopolymers could support efficient uptake and transient gene expression in intact plant tissues [87]. Mechanistically, insights from animal systems show that delivering pre-assembled Cas9 RNPs via nanoparticles enhances cellular uptake and promotes endosomal escape. This method enables rapid, transient genome editing with significantly fewer off-target effects compared to traditional plasmid delivery [88]. These principles are directly applicable to advancing plant genome engineering. Because CRISPR editing only requires Cas nucleases to reside in the nucleus temporarily, biodegradable nanocarriers are particularly advantageous. This short-lived expression is sufficient to create stable, heritable mutations while avoiding the permanent integration of foreign DNA. Such transgene-free approaches align with both regulatory requirements and public preferences for edited crops [75,77]. This technology is especially promising for maize improvement. Traditional methods, such as Agrobacterium-mediated transformation and particle bombardment, often suffer from low efficiency and strong genotype dependence. Plant-based nanocarriers provide a route to overcome these barriers by enabling “DNA-free” delivery of RNPs directly into embryos, leaves, or pollen [75].

By supporting in planta editing, these platforms bypass lengthy tissue culture steps. This not only reduces somaclonal variation but also accelerates the fixation of alleles for drought, heat, and disease tolerance in elite maize germplasm [75,77]. Additionally, these delivery systems can be engineered for high tissue specificity and stimulus-responsive cargo release, such as pH- or redox-triggered mechanisms. This creates a flexible toolbox for climate-smart maize breeding [77]. While current research is in the early experimental stages, the biodegradability and safety of plant-derived nanocarriers make them strategically vital for the future of maize biotechnology.

#### 3.6.2. Plant-Based Selenium and Zinc Nanoparticles for Stress Mitigation683

Plant-derived selenium nanoparticles (SeNPs) and zinc-based nanomaterials, such as ZnSe quantum dots (ZnSe QDs), are emerging as potent tools for enhancing crop resilience by reinforcing antioxidant systems and modulating defense signaling [89,90]. In maize, foliar application of ZnSe QDs at the critical tasselling stage effectively mitigates drought-induced oxidative damage. This is achieved by increasing the activities of key antioxidant enzymes, such as catalase (CAT) and peroxidase (POX)—by up to 98% and 85%, respectively—while concurrently reducing hydrogen peroxide (H_2_O_2_) and malondialdehyde (MDA) levels [91]. These physiological adjustments alleviate lipid peroxidation and preserve membrane integrity, resulting in a 25% increase in photosynthetic rate even under water-deficit conditions. Furthermore, ZnSe QDs improved plant water-use efficiency, which ultimately contributed to a 42% increase in seed number per cob and a 26% gain in grain yield compared to water-sprayed controls [91]. Beyond abiotic stress, SeNPs exhibit potent antimicrobial activity and elicit host resistance against phytopathogenic bacteria and fungi [92,93]. They regulate defense-responsive genes and activate key components such as β-1,3-glucanase to bolster plant immunity. Notably, biogenic SeNPs derived from *Trichoderma* species have been reported to inhibit mycotoxin biosynthesis, offering a significant advantage for food safety in cereal production [92]. Similar combined effects of Se and Zn nanoformulations in mitigating salinity and heavy metal stress have been reported across various crops, driven by the coordinated regulation of redox homeostasis and hormonal balance [94,95]. When synthesized using plant extracts rich in polyphenols or proteins, these nanoparticles exhibit improved biocompatibility and lower toxicity compared to conventional formulations [75,87]. Collectively, plant-derived Se and Zn nanoformulations represent a promising sustainable strategy to buffer maize against the frequent drought and combined stress scenarios projected under climate change.

### 3.7. Rhizosphere Engineering and Microbiome-Informed Breeding

Beyond host-centric traits, multi-omics approaches are increasingly revealing that maize climate resilience is strongly shaped by its root-associated microbiome and the chemistry of root exudates that select and sustain beneficial microbes in the rhizosphere [96]. Root exudation profiles—comprising sugars, amino acids, organic acids, and secondary metabolites—are substantially altered under drought and heat, and these shifts can restructure rhizosphere communities and enrich drought-adaptive taxa such as *Streptomyces* and plant growth-promoting rhizobacteria (PGPR). Experimental work further shows that drought-conditioned microbiomes and specific exudate signatures promote deeper rooting, improved soil water retention, and enhanced osmotic adjustment, leading to measurable gains in drought performance in subsequent plant cohorts [97,98]. These insights have stimulated the concept of “rhizosphere engineering,” in which breeding and genome editing are used to modulate root traits and exudation patterns that recruit nitrogen-fixing or stress-alleviating microbes [99]. For example, associative nitrogen-fixing bacteria isolated from maize rhizospheres, such as *Enterobacter asburiae* strains ASD-07 and ASD-28, can partially substitute mineral nitrogen fertilizers while maintaining or even increasing grain yield, suggesting that host genotypes optimized for their recruitment could simultaneously improve drought resilience and nitrogen use efficiency [100]. Likewise, field and greenhouse studies combining PGPR/PGPB inoculation with variation in maize root traits report that improved nitrogen use efficiency and yield stability are linked to shifts in rhizosphere bacterial communities and root system architecture. More broadly, functional genomics and GWAS are beginning to link specific plant genes controlling organic acid, coumarin, or hormone exudation with predictable changes in rhizosphere community composition and microbial functions, including biological nitrogen fixation, phosphorus solubilization, and induced systemic resistance. Looking forward, targeted editing of maize genes involved in root architecture, mucilage production (e.g., brace-root mucilage that supports colonization by nitrogen-fixing diazotrophs), and exudate biosynthesis offers a powerful route to “program” the rhizosphere toward beneficial consortia that buffer plants against water and nutrient stress [101]. Integrating these host genetic levers with inoculation strategies using carefully selected PGPR consortia, and monitoring outcomes via metagenomics and metabolomics, could enable microbiome-assisted breeding pipelines in which maize genotypes are evaluated not only for intrinsic drought tolerance but also for their capacity to assemble resilient, nitrogen-efficient rhizosphere microbiomes under fluctuating moisture regimes [102].

## 4. Advanced Biotechnological Strategies for Precision Maize Breeding

**Figure 7:**
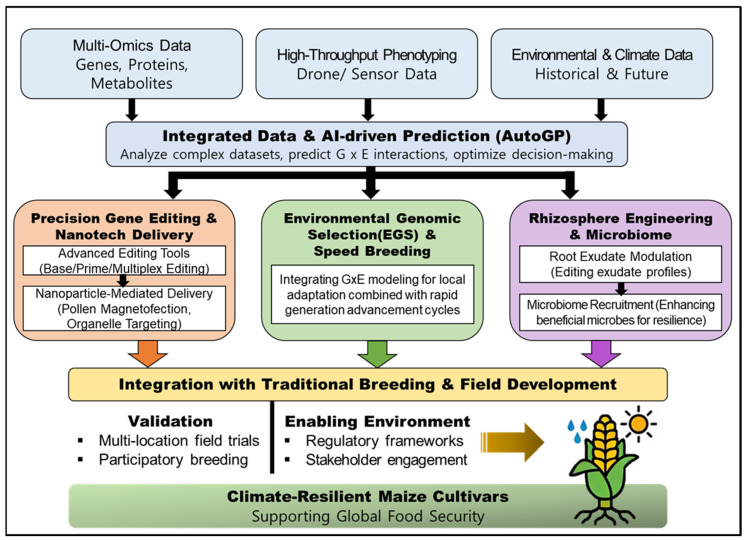
Integrated omics-driven framework for precision maize breeding under climate change, The workflow illustrates how diverse data streams—including multi-omics, high-throughput phenomics, and environmental data—are synthesized through AI-driven prediction models (e.g., AutoGP). This intelligence guides three interconnected strategic pillars: (1) precision genome engineering; (2) environmental genomic selection (EGS) enabled by speed breeding; (3) rhizosphere microbiome modulation. Ultimately, these advanced technologies converge with traditional breeding pipelines to deliver climate-resilient cultivars for global food security. In the diagram, arrows depict the directional flow of data integration and application, while distinct color blocks categorize the different breeding strategies and data layers.

To achieve rapid and precise improvements in maize resilience under changing climates, modern breeding programs are increasingly integrating sophisticated biotechnological tools. Among these strategies, advanced omics technologies are pivotal, facilitating a paradigm shift from empirical phenotype-based selection to data-driven approaches relying on high-dimensional data layers. As illustrated in Figure 7, this integrated framework synthesizes diverse data streams—including phenomics, genomics, transcriptomics, proteomics, and metagenomics—through AI-driven prediction models to guide strategic breeding decisions. This multi-layered approach allows researchers to dissect the complex regulatory networks governing stress responses, thereby accelerating the development of superior genotypes [17].

### 4.1. High-Throughput Phenomics: Accelerating Trait Screening

Traditional crop phenotyping has long acted as a primary bottleneck in breeding pipelines due to its labor-intensive nature, high costs, and low throughput [103]. To overcome these limitations, High-Throughput Phenomics (HTP) technologies offer an innovative alternative by utilizing unmanned aerial vehicles (UAVs) and various ground-based platforms to monitor breeding populations non-destructively. Equipped with RGB, multispectral, hyperspectral, and thermal sensors, these platforms enable the large-scale, real-time capture of key morphological and physiological traits such as leaf area index (LAI), canopy temperature, vegetation indices, and chlorophyll fluorescence [104]. The automated collection of high-resolution temporal data via HTP facilitates rapid screening for drought and heat tolerance traits, ultimately addressing the challenge where genetic gain was previously constrained by the scarcity of phenotypic data [103].

### 4.2. Genomics and Transcriptomics: Precision Gene Discovery

Advancements in genomics have expanded beyond the use of single reference genomes to pan-genome analyses, playing a crucial role in characterizing structural variations (SVs) and presence/absence variations (PAVs) that are often missed by standard re-sequencing methods [17]. High-density SNP markers derived from these essential genetic resources enhance the predictive accuracy of QTL mapping, genome-wide association studies (GWAS), and genomic selection (GS), thereby enabling the early selection of superior lines [17,26]. Concurrently, transcriptomics—particularly RNA sequencing (RNA-seq)—has become an indispensable tool for capturing dynamic gene expression changes under specific stress conditions [23,28]. Recently, the introduction of single-cell RNA sequencing (scRNA-seq) has allowed for the precise analysis of minute stress responses specific to cell types within root and leaf tissues, which bulk sequencing might obscure [105]. The integrated application of these genomic and transcriptomic tools is essential for identifying precise candidate genes and cis-regulatory modules targeted for marker-assisted selection and genome editing [17].

### 4.3. Proteomics, Metabolomics, and Metagenomics: Functional Biomarkers

While genes provide the instructions, proteins and metabolites execute stress adaptation at the functional level. Quantitative proteomics reveals changes in protein abundance and post-translational modifications that do not always correlate with mRNA levels, thereby providing deeper insights into active stress-signaling and defense pathways [106]. In maize, proteomic comparisons between drought-tolerant and susceptible genotypes have identified differential accumulation of molecular chaperones, antioxidant enzymes, transporters, and cell-wall proteins associated with improved stress tolerance [106,107]. Metabolomics complements these analyses by profiling chemical fingerprints—such as compatible solutes (proline, soluble sugars) and secondary metabolites—that serve as direct metabolic biomarkers for selecting resilient lines under drought and heat stress [35,108]. In parallel, metagenomics characterizes the composition and functional potential of the maize rhizosphere and endosphere microbiomes, revealing how specific microbial consortia contribute to nutrient acquisition, hormone modulation, and systemic resistance against abiotic and biotic stresses [100,101]. This knowledge underpins emerging strategies for rhizosphere engineering and the deployment of microbial biostimulants as complementary tools to genetic improvement in climate-resilient maize systems [98,99].

### 4.4. Multi-Omics Integration

To move beyond correlations and more rigorously infer causal mechanisms underlying stress tolerance, breeding strategies are increasingly adopting systems biology frameworks based on multi-omics integration. While single-omics layers provide only partial snapshots, the coordinated integration of genomics, transcriptomics, proteomics, metabolomics, and phenomics enables the construction of comprehensive gene regulatory and metabolic networks (GRNs) that link allelic variation to downstream molecular and physiological responses [109]. This holistic perspective allows researchers to dissect the flow of biological information from genetic variation to phenotypic expression and to pinpoint regulatory bottlenecks amenable to intervention [21,109]. For instance, recent studies in maize have integrated transcriptome and metabolome data to identify key regulatory modules controlling drought responses, revealing complex crosstalk between hormone signaling pathways and stress-associated metabolite biosynthesis that was not apparent from single-layer analyses alone [110]. Such integrated networks serve as high-resolution maps for prioritizing hub genes and metabolites—central nodes whose perturbation can confer broad-spectrum stress resilience with minimal penalties on yield-related traits [22,111].

### 4.5. Artificial Intelligence and Machine Learning in Breeding

#### 4.5.1. Deep Learning and Genomic Prediction

The exponential growth of biological data necessitates advanced computational tools to extract meaningful patterns. Artificial Intelligence (AI) and Machine Learning (ML) algorithms, particularly Deep Learning (DL), are increasingly revolutionizing predictive breeding by modeling complex, non-linear interactions among genotypes, environments, and management factors. Unlike traditional linear models, DL architectures such as Convolutional Neural Networks (CNNs) and Recurrent Neural Networks (RNNs) can process heterogeneous and partially unstructured data. In maize, hybrid CNN-RNN models have demonstrated the ability to capture spatiotemporal dependencies, thereby significantly improving yield prediction accuracy compared to classical methods [112]. Furthermore, recent studies have successfully deployed deep regression models, such as Long Short-Term Memory (LSTM) networks, to predict maize yield using meteorological and environmental features, demonstrating the model’s capability to handle limited farm-level data effectively [113]. Successful implementations in other cereals further illustrate the broader potential of these technologies. In wheat, DL models applied to high-resolution color imagery have achieved high accuracy in detecting stress-related phenotypes and diseases such as Fusarium head blight [114]. These image- and data-informed DL pipelines are now being adapted for large-scale maize phenotyping, particularly for stress-resilience traits that are difficult to capture through conventional field scoring.

#### 4.5.2. Molecular Docking and in Silico Screening

Beyond prediction, computational structural biology—particularly molecular docking—is emerging as a powerful tool for identifying novel stress-related targets and chemical biostimulants. Molecular docking simulates the interaction between small molecules (ligands) and target proteins, enabling in silico screening and optimization of candidate agrochemicals before costly field validation. A major limitation in maize has historically been the scarcity of experimentally solved protein structures compared to model species. However, the advent of AI-driven protein structure prediction tools, such as AlphaFold 2 and the recently released AlphaFold 3, has revolutionized this landscape. These tools allow breeders to predict high-confidence 3D structures of maize stress-responsive proteins (e.g., ZmPYLs, ZmSnRK2s) solely from sequence data, effectively bypassing the bottleneck of experimental crystallography [115,116].

Pioneering work in related crops provides a clear roadmap for adapting these workflows to maize. For example, structure-guided virtual screening was utilized to design ‘Opabactin,’ a synthetic ABA agonist that significantly enhanced drought tolerance in Arabidopsis, wheat, and tomato by precisely modulating receptor activity [117]. Furthermore, the integration of “Deep Docking”—which combines deep learning with physical docking simulations—now enables the rapid screening of ultra-large chemical libraries containing billions of compounds to identify novel biostimulants [118]. By replicating these strategies, researchers can accelerate the dual discovery of stress-managing chemicals and engineered alleles. In parallel to chemical discovery, advances in rational protein design have enabled the creation of ‘made-to-order’ immune receptors; for instance, the engineering of synthetic NLRs fused with nanobodies has provided a blueprint for designing novel genetic targets against evolving pathogens [119]. Integrating such computational strategies into maize breeding will facilitate the rapid development of both chemical biostimulants and engineered traits for durable climate resilience.

### 4.6. Integration of Biotechnological Advances for Tangible Outcomes

The diverse array of biotechnological strategies discussed in this section—ranging from high-throughput phenomics and multi-omics discovery to AI-driven prediction and molecular docking—ultimately converges on a singular goal: the precision identification and manipulation of genetic targets governing stress resilience. While emerging tools like AlphaFold and Deep Docking accelerate the discovery phase, translating these findings into field-ready cultivars remains the critical measure of success. To provide a comprehensive overview of this translation, Table 3 synthesizes key genetic loci, engineered genes, and genomic targets that have successfully enhanced climate resilience in maize. This summary highlights the practical impact of integrating genomic discovery with precision engineering (e.g., *ZmARGOS8* editing) and selection methodologies, offering a proven roadmap for future trait improvement.

## 5. Future Perspectives and Research Directions

### 5.1. Emerging Technologies and Innovations

The next wave of climate-resilient maize improvement will be driven by convergent advances in genome writing, predictive breeding, and high-throughput phenotyping. Prime editing is extending the editable landscape beyond conventional CRISPR-induced indels by enabling programmable base substitutions and precise insertions without inducing double-strand breaks or requiring exogenous donor templates. While efficiency has traditionally been a bottleneck, recent innovations are rapidly overcoming this limitation. For instance, optimizations in prime editor architecture have been proven to significantly enhance editing efficiency in model systems [123]. Importantly, these strategies are being successfully translated to crops, where optimized editor systems (e.g., ePE5max) and engineered pegRNAs have been shown to efficiently generate heritable mutations in maize [124]. To further expand the scope of editability, recent research emphasizes that chromatin accessibility is a critical determinant of editing success, suggesting that epigenetic manipulation can be leveraged to boost efficiencies in recalcitrant genomic regions. Complementary developments, such as epigenome editing to tune stress-responsive networks and improved Cas variants, are widening the scope of engineerable traits for drought and heat resilience [17,77]. Simultaneously, Haploid Induction-Mediated Genome Editing (HI-Edit) is emerging as a powerful pipeline to couple editing with doubled-haploid technology, significantly shortening breeding cycles [57,125]. These biological advances are increasingly integrated with digital technologies; AI-assisted genomic prediction platforms like AutoGP now assimilate genomic, phenomic, and environmental data to accelerate selection under variable climates [126]. When combined with UAV-based multi-scale phenomics, these tools provide the mechanistic anchors needed to bridge genotype–phenotype gaps in complex stress scenarios [103,127].

### 5.2. Integration of Multiple Biotechnological Approaches

Future success will depend not on isolated tools, but rather on the systematic integration of multiple biotechnologies. The synergistic combination of genomic selection (GS) and precision gene editing is becoming a dominant paradigm: GS identifies optimal genetic backgrounds, while editing introduces targeted improvements for large-effect loci [17]. This multi-trait optimization must be supported by advanced delivery frameworks. Nanomaterial-enabled systems are highlighted as promising solutions to overcome transformation bottlenecks; specifically, high aspect ratio nanomaterials have been proven to enable the delivery of functional genetic material into mature plant cells without transgene integration [65,75]. Consequently, integrated pipelines that connect AI-driven target prioritization, rapid validation via speed breeding—a powerful tool to accelerate crop research [128]—and precise delivery will be essential to stack stress tolerance, yield stability, and grain quality in a coordinated manner.

### 5.3. Global Collaboration and Knowledge Sharing

Ensuring global food security through climate-resilient maize requires unprecedented levels of international collaboration. Complex climate challenges demand coordinated efforts to mobilize expertise and resources across borders [17]. International research networks are vital for facilitating the open exchange of genomic data, germplasm, and breeding informatics. Notably, CIMMYT’s contributions to maize improvement have been pivotal in mobilizing these resources for the Global South [6]. Open-access biotechnology initiatives are particularly crucial for lowering entry barriers in low- and middle-income regions, ensuring that innovations like CRISPR and predictive breeding reach the most vulnerable farming communities [77]. Furthermore, capacity-building programs and participatory platforms (e.g., OFAB Africa) that involve local scientists and small-holder farmers are essential to ensure that technological advances translate into tangible, on-the-ground benefits and are equitably distributed [89].

### 5.4. Policy and Regulatory Evolution

The practical deployment of these innovations will be profoundly shaped by evolving governance frameworks. Risk-proportionate regulatory models—which differentiate targeted, transgene-free edits from conventional GMOs—are increasingly adopted to streamline oversight and reduce approval costs for elite edited varieties [17,18]. However, significant challenges remain. Regulatory asymmetry and divergent data requirements across jurisdictions create substantial uncertainty, effectively acting as non-tariff trade barriers [129]. Furthermore, without international harmonization, the prohibitive costs of meeting heterogeneous regulatory standards could restrict market access and stifle innovation, particularly for public sector breeders and SMEs [129]. Consequently, the global synchronization of these standards remains a critical priority to prevent trade disruptions and ensure equitable access to these technologies. In parallel, the safety and environmental lifecycle of emerging tools, such as nanomaterials, must be rigorously evaluated against global biosafety standards [75]. Ultimately, widespread adoption hinges on public trust. Transparent science communication and inclusive governance that engage farmers, consumers, and civil society in decision-making are indispensable for fostering social acceptance and ensuring the responsible deployment of climate-resilient maize [17,77].

## 6. Conclusions

The development of climate-resilient maize represents both a scientific frontier and a global imperative in an era of intensifying climate variability. This review highlights that the strategic integration of advanced biotechnological platforms—spanning CRISPR-based genome editing, Genomic Selection (GS), and multi-omics analytics—offers a transformative route to sustaining productivity under drought and heat stress. By targeting causal alleles and regulatory networks directly, these precision tools are enabling a fundamental paradigm shift from empirical phenotypic selection to a predictive, mechanism-informed breeding framework. Substantial progress has already been realized, as exemplified by proof-of-concept breakthroughs such as the promoter editing of ZmARGOS8 for drought tolerance and the modification of ZmHKT1 for salinity resilience. However, significant bottlenecks remain. The translation of these innovations from laboratory to field is often hindered by genotype-dependent transformation efficiencies and complex genotype-by-environment (G × E) interactions. Overcoming these barriers necessitates parallel innovations in genotype-independent delivery systems—particularly nanotechnology-mediated carriers—and multiplex editing strategies capable of stacking resilience traits into elite germplasm. Looking ahead, the next generation of maize improvement will be defined by the fusion of Artificial Intelligence (AI), structural biology (e.g., AlphaFold), and high-throughput phenotyping. These data-driven technologies are poised to accelerate the discovery and introgression of favorable alleles at unprecedented speeds. Ultimately, the success of biotechnology-driven maize improvement will depend not only on technical convergence but also on harmonized regulatory frameworks and equitable global collaboration. Ensuring transparent risk assessment and inclusive access to these innovations is essential to building truly resilient agricultural systems capable of securing food for future generations.

## Figures and Tables

**Table 1:** Summary of major abiotic and biotic stresses affecting maize production under climate change.

Stress Category	Stress Factor	Morphological & Physiological Impacts	Biochemical & Molecular Mechanisms	References
Abiotic	Drought stress	Stunted vegetative growth and leaf area reduction; Extended Anthesis–Silking Interval (ASI); Severe yield loss (>50% during flowering)	ABA accumulation and stomatal closure; Osmotic adjustment (proline, soluble sugars); Upregulation of antioxidant enzymes (SOD, POD); Expression of drought-responsive genes	[11,12,35]
	Heat stress	Reduced pollen viability and kernel set; Disrupted reproductive physiology at >33–36 °C; Yield reduction (1–1.7% per 1 °C rise above 30 °C)	Oxidative damage to photosynthetic apparatus; Synergistic negative effects with water deficit; Induction of heat-shock response pathways	[2,9,12]
	Combined drought and heat	Accelerated leaf senescence; Greater depression of photosynthesis than single stress; Critical yield penalty	Aggravated lipid peroxidation (MDA accumulation); Complex hormonal signaling crosstalk; Impaired metabolic integrity	[11,12]
	Chilling/low temperature	Poor germination and emergence; Reduced early root and shoot growth, chlorosis; Reduced photosynthetic efficiency and delayed development	Membrane rigidification, ROS accumulation and activation of antioxidant systems; Altered expression of cold-responsive genes and lncRNAs; Changes in photosynthetic and phosphorylation-related transcripts	[28,29,30]
	Flooding/waterlogging	Reduced stand establishment, root swelling and decay, lodging, impaired nutrient uptake and stunted shoots	Root hypoxia, shift to anaerobic metabolism, accumulation of toxic intermediates, perturbation of nutrient and hormone balances, enhanced susceptibility to root diseases	[10,30]
Biotic	Insect pests (e.g., *Spodoptera frugiperda*)	Expansion of overwintering ranges into new regions; Intensified physical damage to canopy and ear; Increased pest generations per year; Defoliation, damaged tassels and ears; Reduced photosynthetic area; Disrupted pollination and increased lodging risk	Trade-off between ABA and JA/SA signaling; Modulation of redox homeostasis by pests; Prioritization of abiotic survival over immune defense	[34,38]
	Fungal pathogens & mycotoxins	Proliferation of *Fusarium* & *Aspergillus* spp.; Increased ear rot incidence; Quality deterioration due to toxin contamination	Biosynthesis of aflatoxins and fumonisins; Interaction with heat/humidity signaling; Co-occurrence of multiple mycotoxins	[41]

**Table 2:** Representative meta-QTLs (MQTLs) associated with drought and yield-related traits in maize.

MQTL ID	Chr.	Position (Mb)	CI (Mb)	Associated Traits	Key Findings & Candidate Genes	References
MQTL1.4	1	158.2–165.5	0.82	ASI, GY, KN	Located near umc11; stable across environments; involves ABA signaling.	[55,56]
MQTL2.2	2	12.5–18.3	0.95	ASI, PH, RL	Includes marker csu133; high pleiotropy for flowering and root traits.	[26,55]
MQTL5.3	5	185.0–192.4	1.1	GY, KN, AD	Significant impact on kernel weight and anthesis date under heat stress.	[55,57]
MQTL6.1	6	142.1–148.8	0.78	ASI, RL, RN	Key hotspot for root-yield co-localization; affects water use efficiency.	[55,56]
MQTL8.2	8	122.5–130.2	1.05	GY, KN, SD	Pleiotropic effects on grain yield and stover digestibility under drought.	[55,58]

Abbreviations: Chr., Chromosome; CI, Confidence Interval; ASI, Anthesis-Silking Interval; GY, Grain Yield; KN, Kernel Number per ear; PH, Plant Height; RL, Root Length; RN, Root Number; AD, Anthesis Date; SD, Stover Digestibility.

**Table 3:** Key genetic loci, engineered genes, and genomic targets contributing to climate resilience in maize.

Approach	Genetic Target	Primary Traits	Key Mechanisms	References
QTL/MQTL	MQTL1.4 (umc11)	Drought Tolerance, ASI	Regulation of flowering synchronization under water stress	[55,56]
	MQTL2.2 (csu133)	Root Architecture, Yield	Coordination of root biomass and yield stability	[26,55]
GWAS	*ZmDREB*, *ZmWRKY*	Seedling Drought Stress	Transcriptional regulation of stress-responsive genes	[57,59]
	*ZmGRAS15*, *ZmDREB2.5A*,	Drought tolerance, root traits	Core regulators of ABA signaling and root development	[59,120]
Genome Editing	*ZmARGOS8*	Drought Tolerance	Promoter replacement (GOS2) for increased expression and yield under drought	[61,62]
	*ZmGDIα*	Disease Resistance (MRDD)	Targeted null mutation for viral disease resistance	[121]
	*ZmCOIα*, *ZmCCT*	Stalk Rot, Drought	Enhanced stalk strength and combined stress tolerance	[15,122]
	*ZmACC1*, *ZmACC2*	Herbicide Resistance	Precision base editing (CBEs/ABEs) for target-site herbicide resistance	[63]
Genomic Selection	Genomic- enabled lines	Water Use Efficiency (WUE)	Long-term genetic gain through GS models	[32,57]

Abbreviations: QTL, Quantitative Trait Locus; MQTL, Meta-QTL; GWAS, Genome-Wide Association Study; ASI, Anthesis-Silking Interval; MRDD, Maize Rough Dwarf Disease; GS, Genomic Selection.

## Data Availability

No new data were created or analyzed in this study. Data sharing is not applicable to this article.
